# Universal Tre (uTre) recombinase specifically targets the majority of HIV-1 isolates

**DOI:** 10.7448/IAS.17.4.19706

**Published:** 2014-11-02

**Authors:** Janet Karpinski, Jan Chemnitz, Ilona Hauber, Josephine Abi-Ghanem, Maciej Paszkowski-Rogacz, Vineeth Surendranath, Debojyoti Chakrabort, Karl Hackmann, Evelin Schröck, María Teresa Pisabarro, Joachim Hauber, Frank Buchholz

**Affiliations:** 1 Medical Systems Biology, Medical Faculty, TU Dresden, Dresden, Germany; 2 Antiviral Strategies, Heinrich Pette Institute – Leibniz Institute for Experimental Virology, Hamburg, Germany; 3 Structural Bioinformatics, TU Dresden, Dresden, Germany; 4 Molecular Cell Biology and Genetics, Max Planck Institute, Dresden, Germany; 5 Clinical Genetics, Medical Faculty, TU Dresden, Dresden, Germany

## Abstract

Current drugs against HIV can suppress the progression to AIDS but cannot clear the patient from the virus. Because of potential side effects of these drugs and the possible development of drug resistance, finding a cure for HIV infection remains a high priority of HIV/AIDS research. We recently generated a recombinase (termed Tre) tailored to efficiently eradicate the provirus from the host genome of HIV-1 infected cells by specifically targeting a sequence that is present in the long terminal repeats (LTRs) of the viral DNA [1]. In vivo analyses in HIV-infected humanized mice demonstrated highly significant antiviral effects of Tre recombinase [2]. However, the fact that Tre recognizes a particular HIV-1 subtype A strain may limit its broad therapeutic application. To advance our Tre-based strategy towards a universally efficient cure, we have engineered a new, universal recombinase (uTre) applicable to the majority of HIV-1 infections by the various virus strains and subtypes. We employed the search tool SeLOX [3] in order to find a well-conserved HIV-1 proviral sequence that could serve as target site for a universal Tre from sequences compiled in the Los Alamos HIV Sequence Database. We selected a candidate (termed loxLTRu) with a mean conservation rate of 94% throughout the major HIV-1 subtype groups A, B and C. We applied loxLTRu as substrate in our established substrate-linked protein evolution (SLiPE) process [4] and evolved the uTre recombinase in 142 evolution cycles. Highly specific enzymatic activity on loxLTRu is demonstrated for uTre in both *Escherichia coli* and human cells. Naturally occurring viral variants with single mutations within the loxLTRu sequence are also shown to be efficiently targeted by uTre, further increasing the range of applicability of the recombinase. Potential off-target sites in the human genome are not recombined by uTre. Furthermore, uTre expression in primary human T cells shows no obvious Tre-related cytopathic or genotoxic effects. Finally, uTre expressing mice show no undesired phenotypes during their normal lifespan. We have developed a broad-range HIV-1 LTR specific recombinase that has the potential to be effective against the vast majority of HIV-1 strains and to cure HIV-1 infected cells from the infection. These results strongly encouraged us in our confidence that a Tre recombinase-mediated HIV eradication strategy may become a valuable component of a future therapy for HIV-infected patients.
